# Essential updates 2021/2022: Update in surgical strategy for perihilar cholangiocarcinoma

**DOI:** 10.1002/ags3.12734

**Published:** 2023-09-08

**Authors:** Fumihiro Kawano, Ryuji Yoshioka, Hirofumi Ichida, Yoshihiro Mise, Akio Saiura

**Affiliations:** ^1^ Department of Hepatobiliary‐Pancreatic Surgery Juntendo University Graduate School of Medicine Hongo, Tokyo Japan

**Keywords:** biliary tract neoplasm, perihilar cholangiocarcinoma, preoperative treatment, treatment strategy

## Abstract

Resection is the only potential curative treatment for perihilar cholangiocarcinoma (PHC); however, complete resection is often technically challenging due to the anatomical location. Various innovative approaches and procedures were invented to circumvent this limitation but the rates of postoperative morbidity (20%–78%) and mortality (2%–15%) are still high. In patients diagnosed with resectable PHC, deliberate and coordinated preoperative workup and optimization of the patient and future liver remnant are crucial. Biliary drainage is recommended to relieve obstructive jaundice and optimize the clinical condition before liver resection. Biliary drainage for PHC can be performed either by endoscopic biliary drainage or percutaneous transhepatic biliary drainage. To date there is no consensus about which method is preferred. The volumetric assessment of the future remnant liver volume and optimization mainly using portal vein embolization is the gold standard in the management of the risk to develop post hepatectomy liver failure. The improvement of systemic chemotherapy has contributed to prolong the survival not only in patients with unresectable PHC but also in patients undergoing curative surgery. In this article, we review the literature and discuss the current surgical treatment of PHC.

## 1 INTRODUCTION

Perihilar cholangiocarcinoma (PHC) was first described in 1965.^1^ Cholangiocarcinoma is a rare tumor arising from the epithelium of the bile duct. It is divided into intrahepatic and extrahepatic cholangiocarcinoma. Extrahepatic cholangiocarcinoma, which can be further sub‐divided into perihilar and distal cholangiocarcinoma based on the anatomical location, accounts for up to 60% of all cholangiocarcinoma.^2^ PHC is commonly classified according to the Bismuth–Corlette classification (BC) based on the extent of proximal biliary infiltration.^3^ BC type 4 PHC extending to the secondary branches of the bile ducts on both sides has been considered a contraindication for resection. However, advances in surgical techniques have allowed for resection to become an acceptable curative treatment option for selected patients with BC type 4 PHC.^4^ Due to a poor understanding of the current classification and relatively common nature of the tumor, misclassification of cholangiocarcinoma subtype may contribute to an underestimation of the incidence of PHC.^5^, ^6^

In most cases of hilar cholangiocarcinoma, right hepatectomy has been the standard procedure for Bismuth type 1/2 PHC because the right hepatic artery runs just behind the hepatic duct. However, recent data show that compared to right hepatectomy, left hepatectomy has a lower postoperative mortality rate and produces similar long‐term results.^7^ It is time to reevaluate the surgical theory relying on actual clinical data.^8^ This review article provides an overview of the surgical treatment of PHC, including new insights from recent publications.

## 2 PREOPERATIVE MANAGEMENT

### 2.1 Preoperative biliary drainage

Most of patients with PHC have developed jaundice when diagnosed. Biliary drainage is recommended to relieve obstructive jaundice and optimize the clinical condition before liver resection.^9^, ^10^, ^11^ Endoscopic biliary drainage (EBD) and percutaneous transhepatic biliary drainage (PTBD) are the preoperative biliary drainage procedures available. Although no consensus has been reached about the preferred approach,^11^ EBD has emerged as the procedure of choice in most centres. Japanese guidelines recommended EBD as the most appropriate procedure in PHC patients.^12^ EBD might result in improved prognosis over PTBD due to the prevention of peritoneal seeding as there is no spillage of bile.^9^, ^13^, ^14^ EBD mainly consisted of endoscopic biliary stenting (EBS) and endoscopic nasobiliary drainage (ENBD). Although EBS has the advantage of less impact for enterohepatic circulation due to internal drainage, high incidence of EBS‐associated cholangitis is considered as problematic especially in Eastern countries.^15^ On the other hand, ENBD also has disadvantages of loss of bile juice and nasopharyngeal discomfort due to external drainage via naso‐pharynx. Takahashi et al. reported the efficacy with inside stent (IS) which is located entirely inside the biliary tree. This report showed that the IS placement provided a more physiological option than ENBD, without nasopharyngeal discomfort and limitations to the patients' life during the waiting time for surgery.^16^

### 2.2 Assessment of liver functional reserve

The preoperative assessment of liver functional reserve is critical to predict the incidence of postoperative liver failure (PHLF). Most patients with PHC are accompanied by biliary stenosis. For these patients, ICG test should be measured after the improvement of jaundice because the results of ICG test sensitively reflect the condition of biliary obstruction and biliary excretory function.^17^, ^18^ ICGK‐F is calculated as plasma clearance rate of ICG functional residual liver volume (FRLV) measured by CT volumetry. Yokoyama et al. found that cut‐off value of ICGK‐F > 0.05 as safe for liver resection for PHC. The risk of PHLF is increased according to the decrement of ICGK‐F. They also found that ICGK‐F, combined pancreatoduodenectomy, the operation time, and blood loss serve as independent risk factors of PHLF and low ICGK‐F as an independent risk factor predicting the postoperative mortality.^19^ In patients with cirrhosis, 99mTc‐GSA uptake corresponds well with ICG clearance test but predicts histological severity better in substantial number of cases. 99mTc‐GSA scintigraphy can be combined with single‐photon emission computed tomography to allow a three‐dimensional measurement of 99mTc‐GSA uptake.^20^ The superiority of these imaging studies is that they can be used to evaluate the liver function of the future remnant liver. Thus, they have been suggested to be useful in patients who have undergone PVE or associating liver partition and portal vein ligation (ALPPS).^21^

### 2.3 Techniques to optimize FRLV

Portal vein embolization (PVE) plays an important role in preventing PHLF after resection of PHC.^22^ In major hepatic resection for PHC, a clear consensus on the cut‐off value of FRLV for indication of PVE has not been reached; however, many reports indicate a FRLV ratio (FRLV/total liver volume (TLV)) of 30%–40% or more as cut‐off value^9^, ^23^ (Table 2). A meta‐analysis conducted by Higuchi et al. reported that 90% of all cases of PHC were indicated for PVE with FRLV/TLV <40%. The safety of PVE is well‐established, with a meta‐analysis by Abulkhir et al. in 2008 showing a complication rate of 2.2% with no deaths among 1088 PVE cases.^24^ Ebata et al. analyzed PVE in 494 cases of biliary tract cancer and reported no deaths or complications requiring special treatment.^25^ Yamashita et al. reported in detail a PVE‐related complication rate of 7.8% (25/319).^26^ In contrast, a case series of two post‐PVE deaths from trisectional PVE was reported. Both patients had cirrhosis and died from sepsis within 1 week after PVE. It is a reminder that caution should be exercised when considering indications, including patient conditions and procedural complexity.^27^ In a report of 16 patients who underwent embolization of the right portal vein + P4 before right trisection, the Nagoya University group reported that hypertrophy of S2 + 3 was significantly greater than that of conventional embolization of the right portal vein alone (122 ± 39 cm^3^ vs. 66 ± 35 cm^3^; *p* < 0.0001) with no complications related to PVE.^28^, ^29^ The MD Anderson Cancer Center also reported that P4 embolization in combination with expanded right hepatectomy resulted in significant S2 + 3 enlargement without an increase in PVE‐related complications.^30^ Yet, some argue that P4 embolization should not be performed due to its high procedural difficulty (high risk of migration of embolized material to the left branch of the portal vein) without an increase in the hypertrophy rate, and the indication should be determined according to the skill level of the interventional radiologist at each institution.^31^

The FRLV increase obtained by PVE has been estimated at 8%–10% at 2–3 weeks after PVE, and, mainly due to disease progression during the waiting period, 10%–15% of non‐resected cases remain after PHC with PVE.^25^, ^26^ Recently, Takahashi et al. reported sequential therapy of PVE followed by systemic chemotherapy for locally advanced PHC, which provided a greater increase in FRLV (median increase rate of 14.4% at median waiting time of 144 days) with acceptable resection rate of 86.6%.^32^ Two techniques, ALPPS and LVD, have been reported as a promising procedure which provides larger kinetic growth of FRLV than the conventional PVE. The former was reported to provide 11 times of extrapolated growth rate than PVE^33^ and the later was reported the median kinetic growth rate of 2.9%/week compared with 1.4%/week of PVE.^34^ In 2012, Schnizbauer et al. reported the associating liver partition and portal vein ligation for staged hepatectomy (ALPPS) technique, which involves ligation of RPV and hepatic dissection to achieve greater FRL enlargement in a shorter period of time.^35^ Olthof et al. reported that ALPPS for PHC was associated with 48% (14/29) 90‐day mortality. Ninety‐day mortality was 13% in 257 patients who underwent major liver resection for PHC without ALPPS. This result implicated that ALPPS was not recommended for PHC.^36^ ALPPS for PHC should be performed at experienced centers after careful consideration of the indications for the procedure.^37^ On the other hand, Sakamoto et al. reported the usefulness of modified procedure of ALPPS for perihilar malignancies, named as partial TIPE ALPPS which consisted of liver partition and trans‐ileocecal portal vein embolization.^38^

To overcome the insufficient remnant liver hypertrophy after PVE, PVE plus hepatic venous embolization (LVD, Liver Venous Deprivation) has recently been performed in parallel with ALPPS. The important point of this procedure is that, unlike ALPPS, it is as safe as PVE alone and produces significantly larger FRL hypertrophy.^34^, ^39^, ^40^ The effect of PVE alone versus PVE plus LVD on liver hypertrophy is currently being investigated in a Phase II RCT in colorectal liver metastasis in France (NCT03841305).^41^

## 3 SURGICAL APPROACH FOR PHC

### 3.1 Standard procedure

The standard procedure for PHC is a hemi‐hepatectomy and caudate lobectomy combined resection with extrahepatic bile duct.^42^, ^43^, ^44^ Depending on the dominance of tumor location, major left‐ or right‐sided hepatectomy is usually selected. The surgical approach is determined based on the patient's condition and residual liver reserve. In cases of poor hepatic reserve, portal vein embolization is preferred, but the criteria for this procedure varies among centers, as do the indications for resection (Table 1).

**TABLE 1:** Previous reports on the procedure for PHC.

Author	Year	Country	No.	Hepatectomy left/right	Trisegmentectomy left/right	PVR (%)	AR (%)	R0 (%)	Morbidity CD > 3 (%)	Mortality (%)
Noji	2016	Japan	209	85/98	15/11	108 (52)	28 (13)	167 (80)	109 (52)	NA
Higuchi	2018	Japan	249	103/113	8/11	56 (22)	19 (8)	162 (65)	92 (37)	9 (4)
Schimizzi	2018	USA	201	79/69	18/25	19 (9)	12 (6)	141 (70)	NA	15 (7)
Jan	2020	Netherland	91	36/45	4/20	81 (89)	5 (5)	59 (65)	48 (53)	10 (12)
Lotte	2021	Netherland	178	76/102	NA	41 (23)	1 (0.1)	56 (31)	103 (58)	24 (14)
Christian	2022	Germany	287	107/180	65/167	164 (92)	5 (1)	122 (43)	186 (65)	42 (15)
Mizuno	2022	Japan	787	440/313	198/66	157 (20)	146 (19)	611 (78)	148 (18)	17(2)

Abbreviations: AR, artery resection; CD, Clavien–Dindo classification; PHC, perihilar cholangiocarcinoma; PVR, portal vein resection.

Right hepatectomy has oncologic and technical advantages over left hepatectomy because (1) the right hepatic artery runs dorsal to the hilar bile duct, (2) the left bile duct is longer than the right bile duct, and (3) the procedure is simpler and portal vein complications can be resected more easily.^8^, ^45^, ^46^ That is why right hepatic resection has been more frequently performed in centrally located PHC.

However, the superiority of right‐sided hepatectomy for PHC is still a contentious issue. Especially considering the larger FRLV, left hepatectomy is more advantageous than right‐sided liver resection, resulting in lower risk of PHLF and postoperative mortality. Franken et al. retrospectively analyzed short‐ and long‐term outcomes of 178 patients who underwent resection of PHC (left‐sided *n* = 76, right‐sided *n* = 102). Postoperative liver failure was more frequent in right‐sided hepatectomy (22% vs. 11%, *p* = 0.052).^47^ As an alternative treatment of choice to right‐sided hepatectomy, Sugiura proposed left hepatectomy with vascular reconstruction.^48^

The indication to perform a trisectionectomy is an important clinical consideration. Trisectionectomy makes it possible to divide the hepatic duct on the limit border. However, it is associated with high risk of postoperative liver failure due to the small FRLV and technical complexity. Compared to right trisectionectomy, left trisectionectomy is the more complex and challenging surgical procedure, and a deep understanding of the anatomy of the portal hepatis is necessary. In particular, left trisectionectomy presents many technical difficulties due to the frequent anatomical variations in the hepatic hilum.^49^ Careful evaluation in preoperative imaging is critical.

### 3.2 Extended surgery for PHC

Innovative surgical techniques can enable us to convert PHC deemed unresectable into resectable PHC. Especially, to achieve the R0 resection, hepatobiliary pancreatic surgeon should acquire a mastery of combined vascular resection and reconstruction for vertical tumor extension and combined pancreaticoduodenectomy for horizontal tumor extension.

#### 3.2.1 Vascular resection

Hilar cholangiocarcinoma easily invades the hepatic artery and portal vein due to their anatomic characteristics precluding R0 resection. Theoretically, combined resection of the infiltrating hepatic artery and portal vein may improve the R0 resection rate and long‐term outcome, and early reports from specialized centers highlight that this is feasible.^50^, ^51^, ^52^, ^53^ Resection and reconstruction of the hepatic artery is considered the more challenging procedure than that of the portal vein.^54^

#### 3.2.2 Hepatopancreatoduodenectomy (HPD)

Simultaneous hepatopancreatic resection is particularly indicated for the treatment of extensive cholangiocarcinoma. The mortality rate after HPD is as high as 8.3%–18.2%.^55^ According to the Japanese national database, the in‐hospital mortality rate after HPD is reported to be 10%, making it the highest‐risk surgical procedure, along with left trisectionectomy.^56^, ^57^ Although the advantage of HPD is a guarantee of negative distal bile duct margin, the indication should be carefully weighed due to its highly invasive nature.

### 3.3 Surgical margin

The incidence of incomplete (R1) resection for PHC still remains high at 10%–72%,^58^, ^59^, ^60^, ^61^ and is a poor predictive factor for survival. The clinical implications of additional resection of the hepatic duct diagnosed intraoperatively as cancer‐positive are still debatable. First, some reports from Western countries have raised concerns regarding the discrepancy between the diagnosis by intraoperative frozen section (IFS) analysis versus permanent histology, resulting in a high false‐negative rate ranging from 10% to 16% in IFS analysis.^62^, ^63^, ^64^ Second, the oncological impact of additional resection is still controversial. Despite the theoretical oncologic advantages, survival data from multiple studies have led to recommendations against re‐resection.^59^, ^62^, ^65^, ^66^ In contrast, other groups reported that R0 resection achieved by additional resection improved prognosis, and they recommend additional resection.^60^, ^67^ This issue has been inconclusive due to the retrospective nature and differences in patient characteristics of the studies listed above. There is also still no consensus on how to treat carcinoma in situ at the margin, which is an issue that needs to be discussed in consideration of the usefulness of an additional resection.^61^ It has been reported that CIS has a worse prognosis than R0 in patients without lymph node metastasis, and that CIS should be avoided, so additional resection may be effective in relatively early‐stage cases without lymph node metastasis.^68^

### 3.4 Outcome for PHC after surgery

Postoperative complications and in‐hospital mortality rates after resection of PHC are the highest among gastrointestinal cancer surgeries even after centralization.^69^ Recent reports indicated a postoperative complication rate of 20%–78%, a severe complication rate of 30.5%–63%, and a postoperative mortality rate of 2%–15% (Table 1). Differences of preoperative and postoperative management policies between regions may affect short‐term outcomes. While it has been well‐known that postoperative complications often negatively affected the prognosis in various cancers, the Nagoya group found postoperative complication have only a small effect in PHC surgery.^70^ A recent report analyzing the U.S. national database suggested that the minimum threshold of ≥7 resections/year resulted in lower 90‐day mortality and improved postoperative outcomes (IP‐weighted OR = 0.49, 95% CI: 0.66–0.87).^71^ According to the Japanese National Clinical Database, postoperative mortality rates for high‐risk HBP surgery have decreased since centralization has been promoted over the past decade.^72^ A benchmark study of 24 high‐volume centers worldwide that performed more than 10 cases per year was also presented. A 90‐day mortality rate of 13% was considered the optimal benchmark for standard hilar cholangiocarcinoma surgery. Surgical outcomes between Western countries and Japan are notably different in this context.^7^, ^73^, ^74^

### 3.5 Liver transplantation (LT)

There have been many reports of LT for PHC, mainly in Europe and the United States.^75^ Initially, due to its unfavorable prognosis, LT was mainly performed for selected patients with a favorable prognosis, such as PHC derived from primary sclerosing cholangitis. Recently, new treatment programs combining neoadjuvant chemoradiation and liver transplantation (NCR‐OLT) and others have reported better outcomes with an overall survival after liver transplantation of 51%–74% and expanded indications for transplantation have been reported.^7^, ^76^, ^77^, ^78^, ^79^ In unresectable PHC, NCR‐OLT confers long‐term survival in highly selected patients able to complete neoadjuvant chemoradiation followed by LT. PSC patients appear to have the most favorable outcomes. There have also been reports of transplantation in resectable PHCC, with results showing a better prognosis compared to resection. However, the report showed that the prognosis of resectable PHC was poorer than in Japan, and the results in the transplant group were consistent with those in Japan, so further reports on transplantation in resectable PHC are needed.^78^, ^80^ A high recurrence rate is of concern when considering extending national graft selection policy to PHC.^81^ LT might have an advantage over resection with respect to liver volume. Clinical issues surrounding donor shortage, immunosuppressive drugs, and patient selection are hurdles to the widespread use of LT.^75^

## 4 NEOADJUVANT THERAPY (NAC)

Neoadjuvant chemotherapy is thought to work both by local control improving the R0 resection rate and by suppression of micro‐metastases improving long‐term survival. However, the evidence for NAC used to treat PHC has not been established to date. Matsuyama et al. reported the efficacy of NAC with Gemsitabin/S‐1 combination therapy on borderline resectable PHC. This study was reported that the overall disease control rate was 91.3% and resection with curative intent was performed for 43 (71%) of the 60 patients. They reported that the median survival time was 50.1 months for the resected patients. For the resected patients, the estimated 3‐year survival rate was 55.8%, and the estimated 5‐year survival rate was 36.4%.^82^ On the contrary, it has been reported that preoperative chemotherapy does not affect prognosis.^83^ A prospective phase III clinical trial on the efficacy of preoperative chemotherapy for cholangiocarcinoma with Gemsitabin/Cisplatin/S‐1 combination therapy is currently underway in JCOG 1920 (jRCTs031200388), the results of which are expected (Table 2).

**TABLE 2:** Previous reports on the cut‐off value for volume of future remnant liver (FRL) in PHC.

Author	Journal	Year	Cut‐off value for PVE	Cut‐off value for PHLF	Cut‐off value for mortality
Ribero D	J Am Coll Surg	2016	‐	FLR < 30%	‐
Bednarsch	HPB	2020	‐	‐	FLR < 40%
Seyama Y	Ann Surg	2003	FLR < 40% (ICGR15 ≤ 10%), FLR < 50% (ICGR15 ≥ 10%)	‐	‐
Yokoyama Y	Br J Surg	2010	FLR < 40%	‐	ICGK‐F < 0.05
Lidsky ME	Ann Gastroenterol Surg	2018	FLR < 40%	‐	
Wiggers JK	J Am Coll Surg	2016	FLR < 30%	‐	FLR < 30%, Incomplete drainage + FLR < 50%
van Gulik TM	Eur J Surg Oncol	2011	FLR < 40%	‐	

Abbreviations: FRL, future remnant liver; PHC, perihilar cholangiocarcinoma; PHLF, post operative liver failure; PVE, portal vein embolization.

## 5 ADJUVANT THERAPY

High‐level evidence for adjuvant therapy in surgery for PHC is lacking due to the small number of cases.^84^, ^85^ Adjuvant capecitabine is recommended by the American Society of Clinical Oncology (ASCO) guideline for patients with resected biliary tract cancer based on the results of BILCAP study, showing a significant improvement of overall survival of 51 months in an adjuvant capecitabine group versus 36 months in the observation group in an intention‐to‐treat analysis.^86^ The ASCOT Trial, which is a Japanese phase III study examining the efficacy of a tegafur‐gimeracil‐oteracil‐potassium combination (S‐1) in postoperative adjuvant therapy in resectable biliary tract cancer, including all types of cholangiocarcinoma, were reported. In an intention‐to‐treat analysis of 440 patients with biliary tract cancer after radical resection, the 3‐year survival rate in the S‐1 group was 77.1%, HR 0.694 (95% CI: 0.514–0.935, *p* = 0.008), compared to 67.6% in the surgery alone group, showing a significant overall survival benefit.^87^, ^88^ S‐1 is currently recommended in Japan as adjuvant chemotherapy after biliary tract cancer surgery including PHC.^87^, ^88^

## 6 CONCLUSION

Here we review recent insights in the surgical treatment of PHC. Hilar cholangiocarcinoma used to be a high‐risk procedure with a high postoperative mortality rate and is relatively rare, with only a few dozen cases per year even in specialized centers. Currently, most results are based on small retrospective cohort studies resulting in low‐quality evidence. To conduct multicenter prospective studies, we need to standardize the surgical procedure.

## AUTHOR CONTRIBUTIONS

Akio Saiura devised the project, main conceptual ideas, and proof outline. Fumihiro Kawano selected and reviewed the references and wrote the manuscript's initial draft. Ryuji Yoshioka, Hirofumi Ichida, and Yoshihiro Mise contributed to the review of the references and assisted with the presentation of the manuscript. All authors have re‐viewed the manuscript.

## FUNDING INFORMATION

The present study was not funded by any organization.

## CONFLICT OF INTEREST STATEMENT

Akio Saiura is a current editorial board member of the *Annals of Gastroenterological Surgery*. The other authors have no conflicts of interest to declare.

## ETHICS STATEMENT

Approval of the research protocol: N/A.

Informed Consent: N/A.

Registry and the Registration No. of the study/trial: N/A.

Animal Studies: N/A.
